# Impact of sarcopenia in elderly patients undergoing elective total hip arthroplasty on postoperative outcomes: a propensity score-matched study

**DOI:** 10.1186/s12871-024-02538-1

**Published:** 2024-04-24

**Authors:** Yan Su, Liangyu Peng, Daoqian Dong, Zhengliang Ma, Xiaoping Gu

**Affiliations:** Department of Anesthesiology, Nanjing Drum Tower Hospital, Affiliated Hospital of Medical School, Nanjing University, Nanjing, 210008 China

**Keywords:** Sarcopenia, Total hip arthroplasty, Prognostic indicator, Postoperative outcomes

## Abstract

**Objective:**

Frailty poses a crucial risk for postoperative complications in the elderly, with sarcopenia being a key component. The impact of sarcopenia on postoperative outcomes after total hip arthroplasty (THA) is still unclear. This study investigated the potential link between sarcopenia and postoperative outcomes among elderly THA patients.

**Methods:**

Totally 198 older patients were enrolled in this study. Sarcopenia in this group was determined by assessing the skeletal muscle index, which was measured using computed tomography at the 12th thoracic vertebra and analyzed semi-automatically with MATLAB R2020a. Propensity score matching (PSM) was employed to evaluate postoperative complications of grade II and above (POCIIs).

**Results:**

The variables balanced using PSM contained age, sex and comorbidities including hypertension, diabetes, hyperlipidemia and COPD. Before PSM, sarcopenic patients with reduced BMI (24.02 ± 0.24 vs. 27.11 ± 0.66, *P* < 0.001) showed higher POCIIs rates (48.31% vs. 15%, *P* = 0.009) and more walking-assisted discharge instances (85.96% vs. 60%, *P* = 0.017) compared with non-sarcopenia patients. After PSM, this group maintained reduced BMI (23.47 ± 0.85 vs. 27.11 ± 0.66, *P* = 0.002), with increased POCIIs rates (54.41% vs. 15%, *P* = 0.002) and heightened reliance on walking assistance at discharge (86.96% vs. 60%, *P* = 0.008).

**Conclusion:**

Sarcopenia patients exhibited a higher incidence of POCIIs and poorer physical function at discharge. Sarcopenia could serve as a valuable prognostic indicator for elderly patients undergoing elective THA.

**Supplementary Information:**

The online version contains supplementary material available at 10.1186/s12871-024-02538-1.

## Introduction

It's crucial to acknowledge that the elderly not only face similar postoperative risks as younger individuals but also contend with age-related functional limitations, including cognitive and physical decline, and frailty. These factors contribute to an increasing trend in postoperative morbidity and mortality rates among the geriatric population [1–3]. Frailty embodies a complex geriatric syndrome described by Dr. Fried and her colleagues, characterized by the presence of three or more indicators: weight loss, exhaustion, diminished physical activity, weakness, and slowness [4]. This intricate phenomenon shares close ties with factors such as sarcopenia, neuroendocrine decline, immune dysfunction, and the potential for unfavorable health consequences [5]. The National Institute on Aging (NIA) suggested that the widespread adoption of the generic term "frailty," encompassing both its conceptualization and measurement, has caused confusion among clinicians and researchers which has, in turn, hindered its incorporation into clinical practices. Additionally, the delayed integration of frailty measurement into clinical routines is likely attributed to a dearth of clinical studies substantiating clear advantages and associated clinical guidelines tailored to the elderly population [6].

**Fig. 1:**
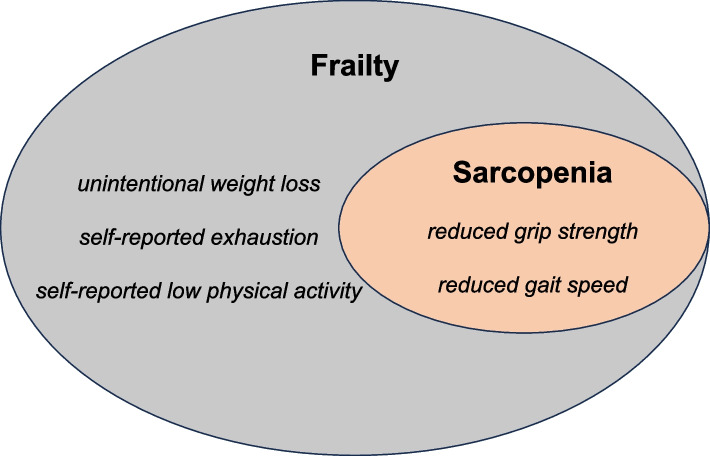
Criteria for the diagnosis of frailty and sarcopenia

Sarcopenia, initially termed by the Rosenberg group in 1989 to depict the reduction in skeletal muscle mass due to aging, comes to our attention for its shared etiological parallels with frailty [7]. In 2018, the EWGSOP (European Working Group on Sarcopenia in Older People) updated their previously proposed comprehensive diagnostic criteria for sarcopenia, which encompass not only diminished muscle mass but also emphasize the importance of muscle strength, and overall muscle function [8]. Within the diagnosis, the grip strength and gait speed highlights sarcopenia’s substantial role within the framework of frailty (depicted in Fig. 1), concurrently serving as predictive markers for cognitive decline among the elderly population [9]. Sarcopenia, being a foundational element of frailty, plays a crucial role in early identification and diagnosis of frailty. For clinical diagnosis of sarcopenia, dual-energy x-ray absorptiometry and bioelectrical impedance analysis are viable, accessible methods for measuring sarcopenia [10]. However, it's well recognized that cross-sectional imaging through computed tomography (CT) or MRI is the most rigorously validated and precise approach to evaluate sarcopenia which is both convenient and effective, facilitates the early identification of elderly patients with sarcopenia and frailty [11, 12]. Remarkably, the occurrence of sarcopenia among community-dwelling elderly individuals had ranged from 9.9% to 40.4% [13]. The changes in human body composition, coupled with underlying frailty, pose significant risks for hospitalized older adults [14] who are particularly vulnerable to adverse outcomes, such as physical disability, diminished quality of life, and mortality [15].

Extensive research had delved into sarcopenia among patients with malignant conditions, revealing a robust connection to postoperative pulmonary complications, infections, readmission rates, and hospitalization duration [16]. The detrimental effects of sarcopenia on patients undergoing cancer and general surgical procedures had been extensively documented [15, 17]. The prevalence of sarcopenia among patients undergoing orthopedic surgery seemed to surpass that of the general population [18]. While the association between sarcopenia and adverse outcomes had been established in multiple surgical and nonsurgical contexts, there is a scarcity of sarcopenia studies specifically focused on orthopedic procedures. Especially in spinal surgeries, varying viewpoints persist regarding the correlation between sarcopenia and adverse outcomes [19, 20]. Reported studies had indicated that elective total hip arthroplasty (THA) can lead to a greater degree of muscle loss compared to that observed in cancer patients. [21, 22]. There is a scarcity of literature addressing the specific impact of preoperative sarcopenia on outcomes following THA. Given the substantial impact of sarcopenia on surgical outcomes and the intricate interplay between bone and muscle, it is highly likely that these implications are further amplified in elderly patients undergoing elective THA. Hence, our objective was to examine whether elderly patients with sarcopenia exhibit an elevated occurrence of postoperative complications following elective THA.

## Methods

### Patients

**Fig. 2:**
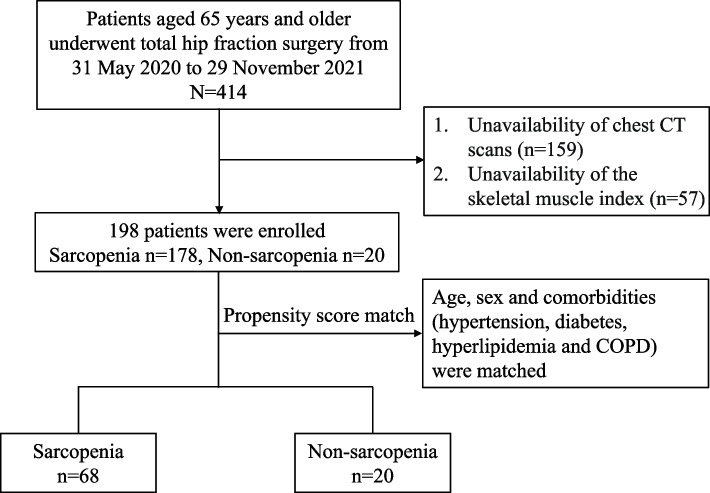
The workflow of patients’ selection

Following the approval of the study protocol by the Institutional Review Board of Nanjing Drum Tower Hospital, Affiliated Hospital of Nanjing University Medical School (IRB No. 2022-765), we conducted a retrospective study at a single center. Between 31 May 2020 and 29 November 2021, patients were required to provide chest CT data upon admission. This requirement aimed to eliminate any potential COVID-19 infections within a 2-week timeframe, thereby minimizing selection bias. We enrolled patients aged 65 years and older who underwent THA during the period. Combining general anesthesia with nerve block anesthesia is a standard procedure in our hospital for these patients. Due to the retrospective nature of the study, informed consent was not obtained. Initially, we enrolled 414 patients; however, 159 were excluded due to the unavailability of chest CT scans in the electronic medical record. Additionally, 57 patients were excluded from the study because of incomplete medical records, leading to an unattainable skeletal muscle index. Ultimately, our study included 198 elderly patients (70 males and 128 females). The patient selection workflow is illustrated in Fig. 2.

### Data acquisition

The data were extracted from electronic medical records and included the following analysis variables: (1) Preoperative characteristics: This encompassed basic demographic data (age, sex, height, body weight, body mass index (BMI)), comorbidities (hypertension, diabetes, hyperlipidemia, chronic obstructive pulmonary disease (COPD), anemia, hypoproteinemia), Barthel index (BI) score, laboratory results, and CT findings. (2) Intraoperative characteristics: This involved anesthesia-related information such as operation time, urine volume, bleeding volume, liquid administration, excessive fluid infusion, blood transfusion, and the application of vasoactive agents. (3) Postoperative characteristics: This included laboratory results, BI score, postoperative length of stay, mode of discharge, and the occurrence of complications.

### Measurement of muscle mass

**Fig. 3:**
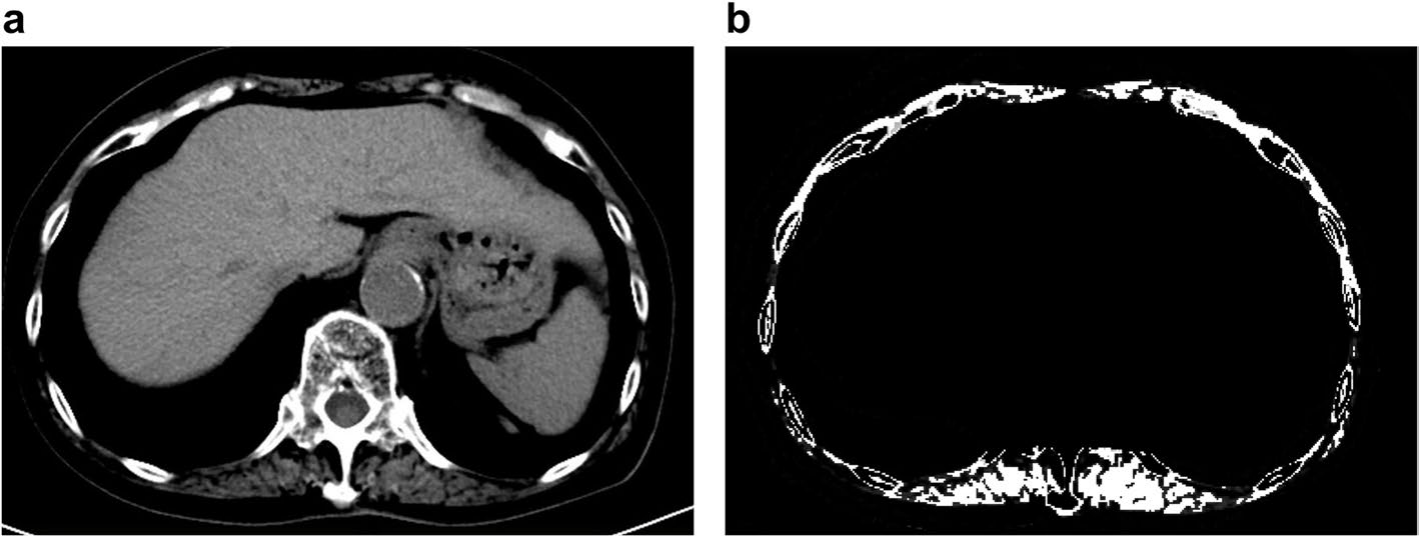
A transverse CT image at the thoracic level T12. **a** The original CT image, **b** Total muscle area was identified by MATLAB, including the erector spinae, latissimus dorsi, external and internal oblique, rectus abdominis, and external and internal intercostal muscles

A single CT image at the 12th thoracic vertebra (T12) was chosen to quantify skeletal muscle characteristics, as this specific anatomical location has a strong correlation with whole-body volume [23]. Based on the standard Hounsfield unit (HU) range, the transverse tissue areas with values ranging from -29 to 150 HU were selected for the analysis of skeletal muscle [24]. All CT images with clearly visible costal processes of T12 were obtained from Neusoft PACS (Neusoft Corp., China) and measured twice using MATLAB R2020a for semi-automatic identification by one trained observer at different times and places who was blinded to the patients' clinical history and postoperative progress. In MATLAB R2020a, the final skeletal muscle area was identified and highlighted and the software also provided the capability to determine the proportion of the highlighted areas in the entire CT image. Finally, the skeletal muscle area for each patient was calculated as the average of the two measurements (Fig. 3). Muscle mass was calculated as the skeletal muscle index (SMI), which is obtained by dividing the total muscle cross-sectional area by the square of height (cm^2^/m^2^). The cutoff values of ≤ 42.6 cm^2^/m^2^ for men and ≤ 30.6 cm^2^/m^2^ for women were used to diagnose sarcopenia in elderly [23].

### Statistical analysis

Variables with a missing data rate exceeding 5% were excluded from the analysis. For variables with missing data below 5%, mean-value imputation was employed to minimize the bias caused by missing values. To balance the potential baseline confounding factors, propensity score matching (PSM) analysis was used to match patients without sarcopenia and those with sarcopenia with a matching ratio of 1: 4 [25, 26]. The “MatchIt” package in R studio (http://www.r-project.org) was used to match the propensity score between two groups, and the matching approach was set as the nearest neighbor algorithm and a caliper value of 0.02 [27]. Histogram plots were built to visualize the distribution of group matching on propensity scores before and after matching and diagnose the quality of matched samples. The variables balanced using PSM contained age, sex and comorbidities including hypertension, diabetes, hyperlipidemia and COPD.

The normality of data distribution was assessed using the Kolmogorov-Smirnov test. Normally distributed data were presented as mean ± standard deviation (SD), while non-normally distributed data were reported as median (interquartile range, IQR). Categorical variables were expressed as counts and percentages (n, %). The analysis of continuous variables used the Mann–Whitney U test or T-test depending on the data distribution. Categorical variables used the chi-square test or Fisher's exact test. A two-sided p-value less than 0.05 was considered statistically significant. All analyses were conducted using IBM SPSS Statistics 26.0 and R studio version 3.6.3.

## Results

### Patients with sarcopenia exhibit a lower BMI

**Fig. 4:**
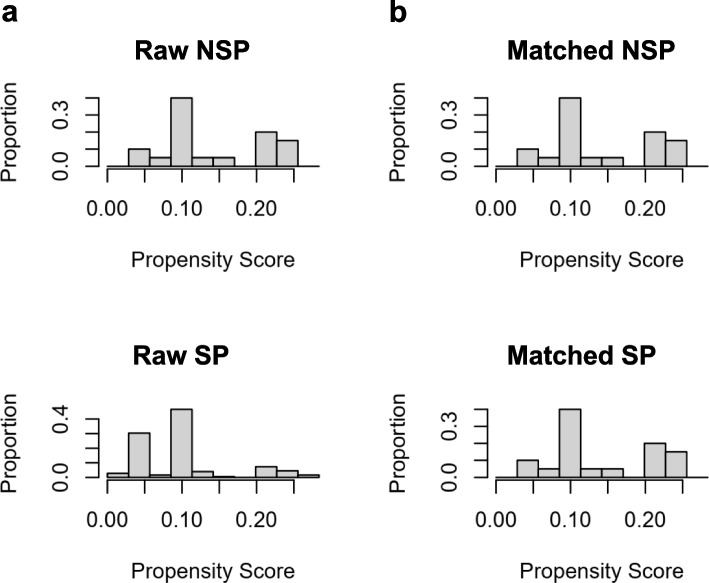
The assessment of sample distribution and covariate balance before and after propensity score matching. **a** Raw unbalanced distribution for both groups before matching. **b** Balanced distribution after the propensity matching

After a rigorous screening process, a total of 198 patients aged 65 and older underwent elective THA and were included in this study. Based on the SMI, the patients were divided into two groups: 178 (89.90%) sarcopenic patients (SP) and 20 (10.1%) non-sarcopenic patients (NSP). We discovered that the balance in matching between the SP and the NSP groups was compromised regarding gender and comorbidities, with age demonstrating a noteworthy influence on both sarcopenia and postoperative complications. Consequently, we incorporated age, gender, and comorbidities into our PSM analysis when comparing these two groups. To mitigate potential confounders and bias, the SP group was meticulously matched with the NSP group in a 4:1 ratio, employing a caliper value of 0.02 which resulted in a well-matched cohort comprising 68 SP and 20 NSP. As portrayed in Fig. 4, the propensity score distribution was graphically displayed before and after PSM, resulting in a transformation from initial imbalance to a harmonized equilibrium closely akin to that of the control group. This visual transformation indicates a successful achievement of well-matched groups. Following rigorous PSM analysis to minimize potential bias, the SP group still exhibited a significant decrease in BMI (23.47 ± 0.85 vs. 27.11 ± 0.66, *P* = 0.002) compared with the NSP group (Table 1). There was a trend toward lower BI scores in patients with sarcopenia (80 (50, 85) vs. 90 (65, 95), *P* = 0.058).

**Table 1:** Baseline characteristics of the raw cohort samples and propensity matched participants

	Unmatched			Matched		
	SP *n* = 178	NSP *n* = 20	*P*	SP *n* = 68	NSP *n* = 20	*P*
Male, n (%)	67 (37.6)	3 (15)	0.05	10 (14.71)	3 (15)	1
Age, n (%)			0.913			0.895
65–75	124 (69.66)	14 (70)		49 (72.06)	14 (70)	
75–85	47 (26.40)	5 (25)		17 (25)	5 (25)	
≥ 85	7 (3.93)	1 (5)		2 (2.9)	1 (5)	
Hypertension, n (%)	103 (57.87)	11 (55)	0.816	38 (55.88)	11 (55)	1
Hyperlipidemia, n (%)	33 (18.54)	8 (40)	0.051	23 (33.82)	8 (40)	0.791
Diabetes, n (%)	30 (16.85)	3 (15)	1	4 (5.88)	3 (15)	0.393
COPD, n (%)	5 (2.81)	0 (0)	1	0 (0)	0 (0)	/
BMI, kg/m^2^	24.02 ± 0.24	27.11 ± 0.66	< 0.001	23.47 ± 0.85	27.11 ± 0.66	0.002
SMI, cm^2^/m^2^	26.07 (23.18, 29.63)	32.63 (31.35, 36.08)	< 0.001	24.78 (22.46, 28.33)	32.63 (31.35, 36.08)	< 0.001
Lymphocyte, 10^9^/L	1.5 (1.1 1.9)	1.65 (1.23, 1.98)	0.504	1.55 (1.2, 1.9)	1.65 (1.23, 1.98)	0.814
Blood platelets, 10^9^/L	206.22 ± 5.18	213.6 ± 16.03	0.653	197.45 ± 13.50	213.60 ± 16.03	0.446
Hemoglobin, g/L	127.70 ± 1.10	130.15 ± 2.16	0.466	130.65 ± 3.68	130.15 ± 2.16	0.907
Blood glucose, mmol/L	4.99 (4.53, 5.82)	4.79 (4.43, 6.46)	0.57	5.12 (4.48, 6.31)	4.79 (4.43, 6.46)	0.402
Blood cholesterol, mmol/L	4.65 (3.96, 5.87)	4.65 (3.87, 5.49)	0.516	4.92 (4.20, 5.45)	4.96 (3.87, 5.49)	0.9
HDL, mmol/L	1.29 ± 0.02	1.27 ± 0.07	0.738	1.33 ± 0.07	1.27 ± 0.07	0.543
LDL, mmol/L	2.73 (2.11, 3.23)	2.91 (2.03, 3.41)	0.858	2.82 (2.19, 3.38)	2.91 (2.03, 3.41)	0.815
Triglycerides, mmol/L	1.13 (0.79, 1.52)	1.59 (1.18, 2.25)	0.002	1.61 (0.97, 2.44)	1.59 (1.18, 2.25)	0.825
Total protein, g/L	65.48 ± 0.41	66.66 ± 1.32	0.363	65.54 ± 0.71	66.66 ± 1.32	0.455
Albumin, g/L	39.2 (37.4, 40.7)	40.05 (39.05, 41.7)	0.038	38.25 (36.80, 41.28)	40.05 (39.05, 41.70)	0.109
CRP, mg/L	5.8 (2.88, 27.43)	3.6 (2.48, 6.08)	0.098	3.95 (2.4, 31.28)	3.6 (2.48, 6.08)	0.783
D dimer	1.14 (0.47, 2.45)	0.66 (0.36, 1.05)	0.008	0.94 (0.33, 1.83)	0.66 (0.36, 1.05)	0.279
Hypoproteinemia, n (%)	30 (16.85)	2 (10)	0.639	13 (19.12)	2 (10)	0.539
Anemia, n (%)	22 (12.36)	0 (0)	0.196	9 (13.24)	0 (0)	0.194
Abnormally high CRP, n (%)	72 (40.45)	3 (15)	0.029	25 (36.76)	3 (15)	0.1
Abnormally high blood glucose, n (%)	33 (18.54)	6 (30)	0.355	9 (13.24)	6 (30)	0.157
BI score	75 (48.75, 90)	90 (65, 95)	0.018	80 (50, 85)	90 (65, 95)	0.058

### Sarcopenia patients exhibit a higher incidence of POCIIs and poorer physical function at discharge

Anesthetic data were meticulously collected, revealing no statistically significant differences between the two groups before and after PSM (Table 2). However, prior to matching, the SP group exhibited higher rates of POCIIs (48.31% vs. 15%) and more instances of discharge with assistance in walking (85.96% vs. 60%) compared to the NSP group. Even after the matching process, the SP group continued to display elevated POCIIs (54.41% vs. 15%) and increased reliance on assistance for walking upon discharge (86.96% vs. 60%). Additionally, the SP group demonstrated lower postoperative hemoglobin levels (106.74 ± 1.70 vs. 112.42 ± 2.22) (all the *P* value < 0.05) when compared with the NSP group (Table 3).

**Table 2:** Intraoperative characteristics of the raw cohort samples and propensity matched participants

	Unmatched			Matched		
	SP *n* = 178	NSP *n* = 20	*P*	SP *n* = 68	NSP *n* = 20	*P*
Surgical methods, n (%)			0.274			0.309
Posterior Lateral Approach	139 (78.09)	19 (95)		54 (79.41)	19 (95)	
Direct Anterior Approach	31 (17.42)	1 (5)		11 (16.18)	1 (5)	
Orthopadisehe Chirurgie Munchen	8 (4.49)	0 (0)		3 (4.41)	0 (0)	
Operation time, minutes	85.00 (70.00, 100)	87.50 (65, 105)	0.724	80.00 (70, 100)	87.50 (65, 105)	0.939
Blood loss, mL	200 (100, 221.43)	200 (162.5, 287.5)	0.389	200 (100, 200)	200 (162.50, 287.50)	0.132
Urine output, mL	300 (200, 500)	400 (300, 575)	0.465	375.00 (212.50, 487.50)	400.00 (300, 575)	0.315
Crystalloid solution, mL	1000 (700, 1500)	1000 (525, 1000)	0.268	1000 (575, 1000)	1000 (525, 1000)	0.567
Colloid solution, mL	500 (500, 1000)	500 (500, 1000)	0.694	500 (500, 500)	500 (500, 1000)	0.192
Excess of intake, mL	1073.90 ± 34.34	1009.50 ± 89.60	0.547	1030.21 ± 57.15	1009.50 ± 89.60	0.859
Blood transfusion, n (%)	20 (11.24)	4 (20)	0.437	9 (13.24)	4 (20)	0.696
Vasoactive agents were used, n (%)	105 (58.99)	12 (60)	1	35 (64.71)	12 (60)	0.613
ASA grade, n (%)			0.755			0.47
II	18 (10.11)	1 (5)		9 (13.24)	1 (5)	
III	152 (85.39)	19 (95)		55 (80.88)	19 (95)	
IV	8 (4.49)	0 (0)		4 (5.88)	0 (0)

**Table 3:** Postoperative characteristics of the raw cohort samples and propensity matched participants

	Unmatched			Matched		
	SP *n* = 178	NSP *n* = 20	*P*	SP *n* = 68	NSP *n* = 20	*P*
Hemoglobin, g/L	108.24 ± 1.03	112.42 ± 2.22	0.188	106.74 ± 1.70	112.42 ± 2.22	0.048
Lymphocyte, 10^9^/L	0.9 (0.7, 1.23)	1.0 (0.73, 1.38)	0.575	0.9 (0.7, 1.38)	1 (0.73, 1.38)	0.966
Blood platelets, 10^9^/L	186.36 ± 4.44	183.94 ± 12.73	0.863	196.07 ± 7.59	183.944 ± 12.73	0.44
CRP, mg/L	63.35 (42.73, 94.85)	44.35 (31.25, 87.13)	0.148	60.50 (43.28, 97.18)	44.35 (31.25, 87.13)	0.162
Hemoglobin decreased compared with preoperative value, g/L	19.46 ± 0.95	17.73 ± 2.19	0.557	18.03 ± 1.73	17.73 ± 2.19	0.93
BI score at discharge	65 (60, 65)	65 (60, 65)	0.637	65 (60, 70)	65 (60, 65)	0.97
Length of postoperative hospital stay, hours	99.17 (77.29, 124.5)	99.58 (73.40, 112.85)	0.26	98.54 (77.67, 142.44)	99.58 (73.40, 112.85)	0.176
Mode of discharge, n (%)			0.017			0.008
Death	2 (1.12)	0 (0)		1 (1.47)	0 (0)	
Without tools	23 (12.92)	8 (40)		7 (10.29)	8 (40)	
With tools	153 (85.96)	12 (60)		60 (86.96)	12 (60)	
POCIIs, n (%)	86 (48.31)	3 (15)	0.009	37 (54.41)	3 (15)	0.002
II (complications treated with drugs)	76 (42.70)	3 (15)		33 (48.53)	3 (15)	
III (surgical site complications treated by reoperation)	2 (1.12)	0 (0)		0 (0)	0 (0)	
IV (with AICU management)	6 (3.37)	0 (0)		3 (4.41)	0 (0)	
V (death)	2 (1.12)	0 (0)		1 (1.47)	0 (0)	

## Discussion

This retrospective study revealed that preoperative sarcopenia stands as a robust prognostic determinant, correlating with increased postoperative complications in elderly patients undergoing elective THA, both prior to and following PSM. Furthermore, sarcopenia displayed a substantial link to decreased ambulation capacity without assistance, as well as increased reliance on aids like wheelchairs and platform wagons upon discharge. Our study illuminates the crucial role of identifying preoperative sarcopenia in the elderly which may help anesthesiologists in promptly recognizing preoperative frailty and optimizing surgical risks in elderly patients.

Sarcopenia is characterized by the progressive loss of muscle mass, strength, and function with aging, which has been investigated for its predictive value in cancer patients over the past few decades [15]. CT scan is a practical and reliable method for evaluating SMI in surgical patients, which has been widely recognized as the gold standard of measurement of human body composition [28]. We opted for skeletal muscle area measurement at the T12 level rather than the more commonly used L3 level, as chest CT scans are more clinically viable and not only facilitate the evaluation of body composition but also enable assessment of respiratory conditions preoperatively. We utilized SMI reference values tailored to the elderly population, accounting for the limitations of our retrospective study. The established T12 cutoff values based on elderly patients can effectively diagnose sarcopenia via chest CT and assess its correlation with outcome parameters in diverse conditions [23, 29].

Based on earlier research, the connection between sarcopenia and postoperative complications in hip surgery remains infrequent and debated. Yoo et al. indicated that hip fracture patients aged 60 years or older with sarcopenia exhibit a 1.8-fold increase in one-year mortality compared to those without sarcopenia. However, the postoperative one-year mortality rate showed no noteworthy distinction between the sarcopenia and non-sarcopenia groups in hip surgery [30, 31]. Sarcopenia has been confirmed to be associated with a heightened risk of postoperative implant-related complications following THA [32, 33], but other complications and elderly population were rarely reported. Previous studies [34, 35] also shown that preoperative sarcopenia affect the recovery of physical function after THA which is potentially related to increased postoperative complications. Research on complications following THA in elderly patients with sarcopenia remains limited.

In this study, the primary outcome was postoperative complications based on the Clavien-Dindo classification, specifically concentrating on grade II and higher which was identified as postoperative complications (POCIIs) in this study. The Clavien-Dindo classification system comprises seven distinct grades, with patients falling into grade II and higher necessitating additional medical interventions [36] which contained anemia, hypoproteinemia, dyspnea, skin allergy, hypertension, hyperglycemia, liver damage, pulmonary infection, seizures, gout attacks, and urinary retention in this study. Our study aimed to confirm POCIIs in elderly patients with sarcopenia undergoing THA. We observed that POCIIs were more than three times higher in elderly sarcopenia patients compared to the non-sarcopenia group, both pre and post PSM. Furthermore, elderly patients with sarcopenia experienced a notable reduction in walking capacity upon discharge, both before and after PSM. The Clavien-Dindo classification categorized postoperative complications in our study with a predominant occurrence of Grade II complications, which was consistent with the findings in geriatric hip fracture patients [37]. Grade II complications, encompassing anemia, hypoproteinemia, dyspnea, skin allergy, hypertension, hyperglycemia, liver damage, pulmonary infection, seizures, gout attacks, and urinary retention, were identified in this study. Anemia independently heightens the risk of sarcopenia in the elderly Chinese population [38]. This correlation partly stems from the fatigue-prone nature, abnormal Iron metabolism and malnutrition of anemic individuals, which can result in reduced physical activity and subsequent muscle function decline [39]. The increased prevalence of postoperative anemia in patients with sarcopenia aligns with previous research finding [40]. Addressing anemia pre and postoperatively may emerge as a dependable strategy for preventing both sarcopenia and POCIIs. Erythropoietin (EPO) was found the most commonly utilized drug to improve anemia in this study. EPO could enhance muscle strength and mass, alleviating sarcopenic symptoms but there existed gender differences [41]. Moreover, it mitigated postoperative infections in hip fracture patients and curtailed hospitalization duration [41] which had potential therapeutic implications and needed further studies.

In terms of physical function, although there was a slight trend towards lower BI scores (*P* = 0.058) in the SP before surgery, our findings indicated a significant decline in ambulatory capacity and an increased reliance on assistive tools upon discharge among individuals with sarcopenia. This indicates a swift deterioration of lower limb strength in sarcopenic patients, exemplifying fragility that could potentially exacerbate over time. In addition to reduced lower limb muscle mass and function, sarcopenia was associated with impaired detrusor contractility and respiratory muscles which could cause urinary retention, dyspnea and pulmonary infection postoperatively [42, 43]. Age-related changes in body composition manifest as an accumulation of fat mass accompanied by a decline in muscle mass, potentially exacerbating the onset and progression of sarcopenia [44]. Both before and after PSM, a noticeable reduction in BMI was evident among sarcopenia patients and a diminished BMI corresponded with an elevated vulnerability to frailty [45], potentially exposing the elderly to unfavorable health consequences.

We aim to raise awareness among clinicians regarding the degenerative changes in motor organs in the elderly through our study on sarcopenia, with the goal of preventing adverse outcomes in elderly patients with sarcopenia post-surgery. Japanese scholars have proposed that the concept of locomotive syndrome can identify individuals expected to develop sarcopenia and frailty, allowing for proactive preventive measures to be implemented at an earlier stage [46, 47]. Interventions aimed at enhancing the nutritional status, muscle strength, and overall function of sarcopenic patients may include strategies such as implementing targeted nutritional supplementation and tailored exercise therapy [48]. Nonetheless, the integration of consistent resistance exercise into clinical application remains a formidable challenge, compounded by the absence of authorized pharmaceutical interventions for the management of sarcopenia at present [49]. Collaboration with surgeons, rehabilitation physicians, and nutritionists is strongly advised for preoperative evaluation and additional medical care in elderly patients with sarcopenia.

The study does possess certain limitations, which necessitates a cautious interpretation of its findings. This study, conducted retrospectively at a single center, employed muscle mass as the exclusive parameter for sarcopenia diagnosis, without assessing muscle strength and function. Two hundred and sixteen patients were excluded due to the absence of muscle mass data. To examine potential selection bias, we compared the preoperative conditions of these excluded patients with those included in the study. No significant differences were found between the two groups in terms of age, gender, or ASA grade (see Supplementary Table). While we have diligently employed PSM analysis to manage potential confounding variables, it remains challenging to entirely eliminate the residual impact of confounding factors. Furthermore, though there existed no discernible disparity in postoperative outcomes among individuals undergoing hip surgery during the COVID-19 pandemic and those treated in non-pandemic periods [50], the potential influence of COVID-19 vaccination on diminishing postoperative complications was not accessible in our study [51]. Prospective large-cohort studies, other surgical types, long-term prognosis and multimodal pre-habilitation in elderly patients should be the following research.

## Conclusion

In summary, elderly patients with preoperative sarcopenia face an increased risk of postoperative outcomes. Enhancing preoperative assessment protocols for elderly patients and multimodal pre-habilitation for sarcopenia may greatly benefit elderly patients undergoing surgical procedures.

### Supplementary Information

**Supplementary Material 1.**

## Data Availability

All data generated or analyzed during this study are included in this article.

## Abbreviations

THA: Total hip arthroplasty
PSM: Propensity score matching
POCIIs: Postoperative complications of grade II and above
NIA: The National Institute on Aging
EWGSOP: European Working Group on Sarcopenia in Older People
CT: Computed tomography
BMI: Body mass index
COPD: Chronic obstructive pulmonary disease
ASA: American society of anesthesiologists
BI: Barthel index
HU: Hounsfield unit
SMI: Skeletal muscle index
SD: Standard deviation
IQR: Interquartile range
SP: Sarcopenic patients
NSP: Non-sarcopenic patients
EPO: Erythropoietin
